# A comprehensive analysis of FBN2 in bladder cancer: A risk factor and the tumour microenvironment influencer

**DOI:** 10.1049/syb2.12067

**Published:** 2023-06-19

**Authors:** Zechao Lu, Zeguang Lu, Yongchang Lai, Haobin Zhou, Zhibiao Li, Wanyan Cai, Zeyao Xu, Hongcheng Luo, Yushu Chen, Jianyu Li, Jishen Zhang, Zhaohui He, Fucai Tang

**Affiliations:** ^1^ Department of Urology The Eighth Affiliated Hospital Sun Yat‐sen University Shenzhen Guangdong China; ^2^ The Second Clinical College of Guangzhou Medical University Guangzhou Guangdong China; ^3^ The First Clinical College of Guangzhou Medical University Guangzhou Guangdong China; ^4^ Department of Social and Behavioural Sciences City University of Hong Kong Hong Kong China

**Keywords:** bioinformatics, cancer, data analysis

## Abstract

Bladder cancer (BLCA) is a common and difficult‐to‐manage disease worldwide. Most common type of BLCA is urothelial carcinoma (UC). Fibrillin 2 (FBN2) was first discovered while studying Marfan syndrome, and its encoded products are associated with elastin fibres. To date, the role of FBN2 in BLCA remains unclear. The authors first downloaded data from The Cancer Genome Atlas (TCGA) and Gene Expression Omnibus (GEO). The patients were divided into high FBN2 expression and low FBN2 expression groups, and the survival curve, clinical characteristics, tumour microenvironment (TME), and immune cell differences were analysed between the two groups. Then, the differentially expressed genes (DEGs) were filtered, and functional enrichment for DEGs was performed. Finally, chemotherapy drug susceptibility analysis based on the high and low FBN2 groups was conducted. The authors found upregulated expression of FBN2 in BLCA and proved that FBN2 could be an independent prognostic factor for BLCA. TME analysis showed that the expression of FBN2 affects several aspects of the TME. The upregulated expression of FBN2 was associated with a high stromal score, which may lead to immunosuppression and be detrimental to immunotherapy. In addition, the authors found that NK cells resting, macrophage M0 infiltration, and other phenomena of immune cell infiltration appeared in the high expression group of FBN2. The high expression of FBN2 was related to the high sensitivity of some chemotherapy drugs. The authors systematically investigated the effects and mechanisms of FBN2 on BLCA and provided a new understanding of the role of FBN2 as a risk factor and TME influencer in BLCA.

## Graphical abstract

We systematically investigated the effects and mechanisms of FBN2 on BLCA and provided a new understanding of the role of FBN2 as a risk factor and TME influencer in BLCA.

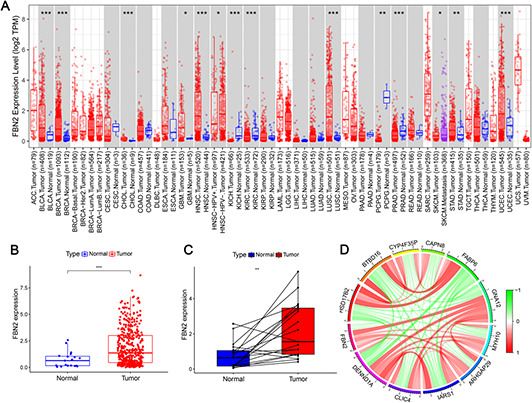

## 1 INTRODUCTION

BLCA is the 10th most common malignancy and the sixth most common malignancy in men, with more than 570,000 new diagnoses and an estimated 210,000 deaths worldwide [1]. The histological type of most BLCA is UC, and non‐muscle invasive bladder cancer (NMIBC) accounts for approximately 75% of BLCA(2). The standard treatment for patients with advanced BLCA is still platinum‐based chemotherapy [3]. For patients with NMIBC, transurethral resection of bladder tumour (TURBT) and follow‐up combined with immunotherapy and chemotherapy are the vital choices to manage [2]. NMIBC is considered an immunotherapy‐responsive cancer type [4], and *Bacillus* Calmette ‐ Guerin (BCG) as immunotherapy is known as a highly effective form of intravesical treatment for NMIBC [5]. However, some of the patients treated with BCG may experience non‐response, recurrence, severe adverse reactions, or remain progress to muscle invasive bladder cancer (MIBC) [6, 7]. Radical cystectomy, even chemotherapy, immune checkpoint blockade (ICB) therapy is often required for MIBC patients. However, there are still some adverse effects of ICB therapy [8, 9, 10].

Tumour cells, stromal cells, and tumour‐infiltrating immune cells make up the majority of the TME [11]. According to research, stromal components can form the TME, impact chemotherapy and immunotherapy response, and encourage cancer progression [12]. To solve the bottleneck of immunotherapy, it is urgent to study the TME of BLCA, which is also the focus of current research.

FBN2 is a 279.57 kb protein‐coding gene mapping in 5q23‐31. The protein that FBN2 encodes is associated with elastin fibres, which are associated with the matrix [13]. Past studies on FBN2 mainly focused on congenital contractural arachnodactyly (CCA), and it has been proven that mutation in FBN2 is an important cause of CCA [14]. Recently, some studies have found a possible link between FBN2 and some types of tumours, including colorectal adenocarcinomas and non‐small‐cell lung cancer [15, 16]. High expression of FBN2 may be associated with TGF‐β signal enhancement [17]. TGF‐β is generally considered to be associated with tumour progression and immunosuppression in the TME [18, 19]. This suggests that FBN2 may be correlated with the TME. It has been found that the epithelial‐mesenchymal transition or TME related gene prognostic models containing FBN2 have good prognostic performance [20, 21]. This implies that FBN2 plays an important role in BLCA. However, the effect of high or low expression of FBN2 on TME and the efficacy of chemotherapy drugs in BLCA remains unclear. We conducted an in‐depth study on the prognostic properties of FBN2 in BLCA and its influence on immune and chemotherapy drugs in order to gain a comprehensive understanding of the role of FBN2 in bladder cancer.

## 2 METHOD

### 2.1 Download of data

All data were downloaded on September 5, 2021. The transcriptome data, and corresponding clinical data of BC patients were downloaded from TCGA (https://tcga‐data.nci.nih.gov/tcga/). After excluding patients without the complete the survival time and survival state from further evaluation, 406 samples of BLCA patients and 19 normal tissues were successfully downloaded from TCGA. And 87 samples of BLCA patients with available clinical information were extracted from GSE19915 on GEO (https://www.ncbi.nlm.nih.gov/geo) for external validation in this study, collecting only the GPL5186 platform (Swegene Human 27K RAP UniGene188 array).

### 2.2 Analysis of FBN2 differential expression

TIMER [22] (http://timer.comp‐genomics.org/) is a powerful web analysis tool that can perform pancancer analysis for the differential expression levels with Wilcoxon testing of particular genes between tumours and adjacent normal tissues based on TCGA, and then box plots are used to visualise the results [22]. To systematically understand the expression levels of FBN2 in different tumours, TIMER was used to analyse the expression levels of FBN2 in different tumours that TCGA contained.

For the expression of FBN2 in BLCA, the R package ‘limma’ [23] was used to analyse and visualise the difference in FBN2 expression between BLCA and normal tissues. The correlation between FBN2 and other genes was explored with the filter criteria set to a correlation coefficient >0.4 and *p* value <0.001. The R package ‘circlize’ [24] was used to draw the association networks of FBN2 and other genes.

### 2.3 Survival analysis based on FBN2 expression

Patients were divided into a high FBN2 expression level group and a low FBN2 expression level group on the basis of the median FBN2 expression. Kaplan‐Meier (KM) analysis was performed for TCGA samples and GEO samples with the end events set to death, and log‐rank tests were performed to determine the *p* value. KM analysis was completed by the R package ‘survival’ (https://CRAN.R‐project.org/package=survival).

The receiver operating characteristic (ROC) curve was used to evaluate the accuracy of FBN2 for predicting prognosis. The area under the curve (AUC) was an effective method to summarise the accuracy of the ROC, and the higher the value of the AUC (no more than 1), the better the predicting ability. The AUCs at 1, 3, and 5 years were calculated. ROC analyses were completed by the R package ‘timeROC’ [25].

### 2.4 Correlation analysis between FBN2 and clinical features

To analyse the relationship between FBN2 expression levels (high FBN2 expression and low FBN2 expression) and clinical features in detail, the R package ‘limma’ [23] was used. The clinical features included in the analysis were age, gender grade, stage, and TNM stage. The R packages ‘ComplexHeatmap’ [26] and ‘ggpubr’ (https://CRAN.R‐project.org/package=ggpubr) were used to visualise the results. To further test whether the FBN2 expression levels and clinical features were independent prognostic factors, univariate and multivariate Cox regression were executed, and forest plots were created to present results visually based on the R package ‘survival’ (https://CRAN.R‐project.org/package=survival).

### 2.5 Screening of DEGs

To explore the underlying mechanism of the difference between the high FBN2 expression group and the low FBN2 expression group, the DEGs between the two groups should be found for follow‐up analysis. The R package ‘limma’ [23] was used to calculate and filter DEGs. The filter threshold was set to |logFC| >1 and FDR (false discovery rate) <0.05. The heatmap was created by the R package ‘pheatmap’ (https://CRAN.R‐project.org/package=pheatmap).

### 2.6 Functional enrichment for DEG

Gene Ontology (GO, http://geneontology.org) is a powerful database of gene functions [27]. The Kyoto Encyclopaedia of Genes and Genomes (KEGG, www.kegg.jp) is also a database containing gene functions and related pathways [28]. To explore the DEG functions, gene function enrichment was executed based on GO and KEGG. For GO enrichment, the analysis was based on the R packages ‘clusterProfiler’ [29], ‘org.Hs.eg.db’, ‘enrichplot’ (https://github.com/GuangchuangYu/enrichplot), ‘ggplot2’ [30], ‘circlize’ [24], ‘RColorBrewer’ (https://CRAN.R‐project.org/package=RColorBrewer), ‘dplyr’ (https://CRAN.R‐project.org/package=dplyr), and ‘ComplexHeatmap’ [26]. For KEGG, the analysis was based on the R packages ‘clusterProfiler’ [29], ‘org.Hs.eg.db’, ‘enrichplot’(https://github.com/GuangchuangYu/enrichplot), and ‘ggplot2’ [30]. To further comprehensively explore the DEG functions, gene set enrichment analysis (GSEA) was performed based on KEGG enrichment by the R packages ‘limma’ [23] and ‘org.Hs.eg.db’. ‘clusterProfiler’ [29], ‘enrichplot’ (https://github.com/GuangchuangYu/enrichplot) and gene annotation file ‘c2.cp.kegg.v7.5.1.symbols.gmt’, which was downloaded from the Molecular Signature database (MSigDB, http://www.broad.mit.edu/gsea/msigdb/).

### 2.7 Analysis of the relationship between FBN2 and the TME

The analysis of the difference in the TME between the high FBN2 expression group and the low FBN2 expression group was based on ESTIMATE, which is a scoring method based on expression data [31]. After using the R packages to obtain the scoring documents, the R packages ‘limma’ [23], ‘reshape2’ [32], and ‘ggpubr’ (https://CRAN.R‐project.org/package=ggpubr) were used to plot.

Then, the differential analysis of immune cells between the high‐low FBN2 expression group was performed based on CIBERSORT [33]. The R packages ‘e1071’ (https://CRAN.R‐project.org/package=e1071), ‘preprocessCore’ (https://github.com/bmbolstad/preprocessCore), and ‘limma’ [23] were used, and the filter condition of the *p* value was set to 0.05. In addition, correlation analysis between the FBN2 expression and immune cells was executed.

Tumour mutation burden (TMB) is considered to predict the therapeutic effect of immune checkpoint blockade (ICB) therapy, TMB has been proven to be a tumour biomarker in some cancers [34]. Based on the R packages ‘limma’ [23], ‘ggplot2’ [30], the relationship between TMB and FBN2 expression was analysed by Spearman correlation analysis.

### 2.8 Chemotherapy drug susceptibility analysis

To evaluate the FBN2 ability in predicting the clinical response of treatment, we calculated the half maximal inhibitory concentration (IC50) of chemotherapeutic agents commonly used for BLCA, including ‘cisplatin’, ‘docetaxel’, ‘doxorubicin’, ‘gemcitabine’, ‘methotrexate’, and ‘vinblastine’. The differential IC50 of chemotherapeutic agents between low and high FBN2 expression groups was predicted by the ‘pRRophetic’ package [35]. Based on gene expression microarray data, the pRRophetic package, which was used to determine IC50 with success in previous research [36, 37, 38, 39], was performed for the prediction of clinical chemotherapeutic response by using statistical models from the gene expression and drug sensitivity data from cell lines in the Cancer Genome Project [40] as a training set.

## 3 RESULT

### 3.1 High FBN2 expression in BLCA tissues

**FIGURE 1:**
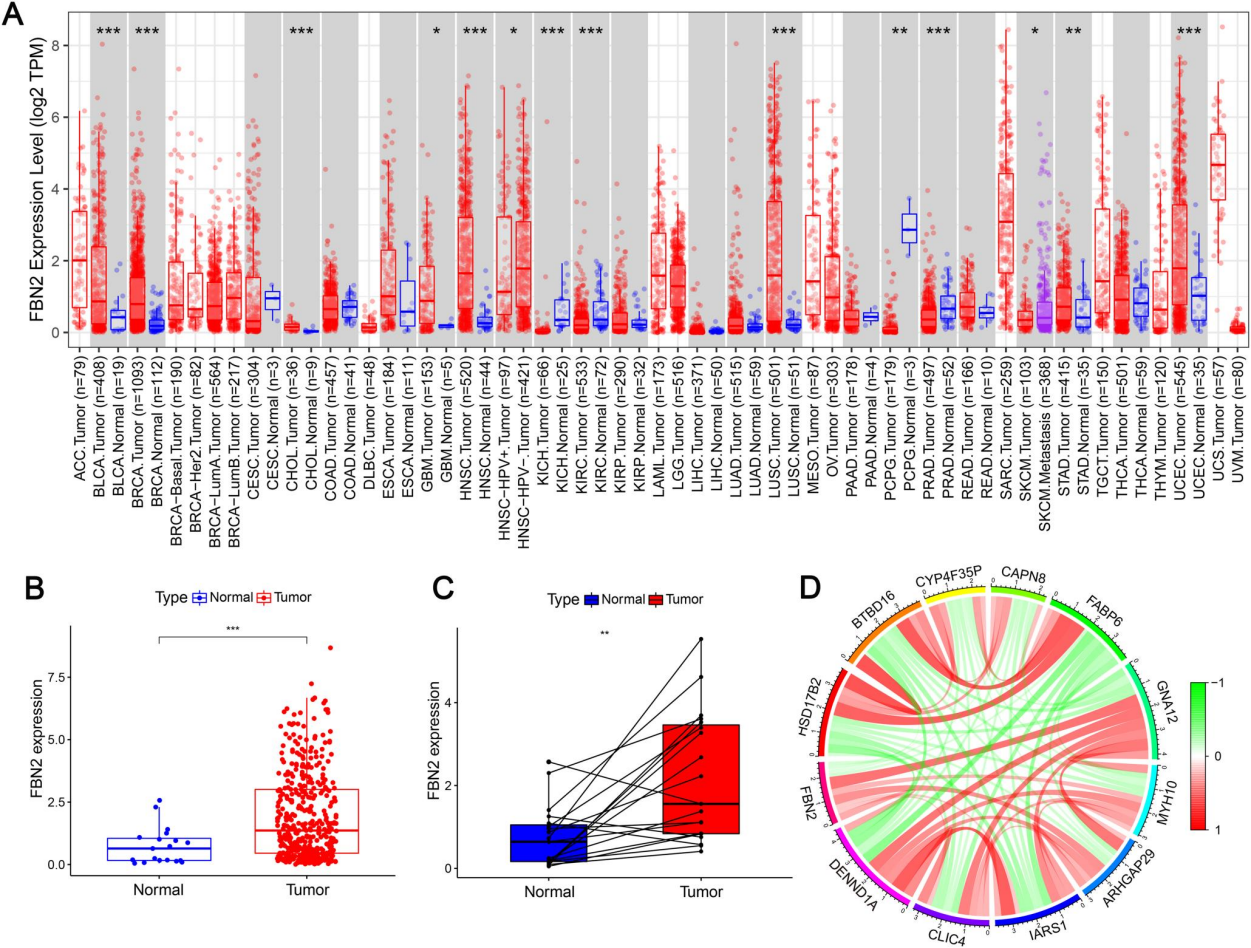
(a) Box plots for pananalysis of tumour and normal tissues. * indicates *P* < 0.05, ** indicates *P* < 0.01, *** indicates *P* < 0.001, and the symbols are common to other figures. (b) Box plot for all tumour samples and normal samples. (c) Box plot for normal tissues and corresponding tumour tissues. (d) Each colour in the outer ring represents a different gene. Between genes, red lines represent positive associations, and green lines represent negative associations. The darker the colour is, the higher the positive or negative correlation coefficient.

The pan‐cancer analysis showed the difference in FBN2 expression between normal tissues and several types of tumours (Figure 1a). Among the tumours, the statistical significance of the difference between tumour and normal tissues involved bladder cancer (BLCA), breast cancer (BRCA), cholangiocarcinoma (CHOL), glioblastoma multiforme (GBM), head and neck squamous cell carcinoma (HNSC), kidney chromophobe (KICH), kidney renal clear cell carcinoma (KIRC), lung squamous cell carcinoma (LUSC), pheochromocytoma and paraganglioma (PCPG), prostate adenocarcinoma (PRAD), stomach adenocarcinoma (STAD), and uterine corpus endometrial carcinoma (UCEC). In these cancers, except for PCPG, PRAD, KIRC, and KICH, FBN2 is highly expressed in tumour tissues, including BLCA. This may suggest that FBN2 is a risk factor in these cancers.

For BLCA, the differential FBN2 expression in all tumour tissues and normal tissues had a statistical significance with a *p* value less than 0.001, and in 19 tumour tissues and corresponding normal tissues also had a statistical significance with a *p* value less than 0.01 (Figure 1 b,c). The correction between FBN2 and other genes was also analysed with a screening condition of a *p* value less than 0.001. The results showed that the expression of 11 genes was related to FBN2 expression (Figure 1d). FBN2 had a positive correlation with GNA12, MYH10, ARHGAP29, IARS1, CLIC4, and DENMD1A and a negative correlation with HSD17B2, BTBD16, CYP4F35P, CAPN8, and FABP6.

### 3.2 High FBN2 expression is associated with low overall survival

**FIGURE 2:**
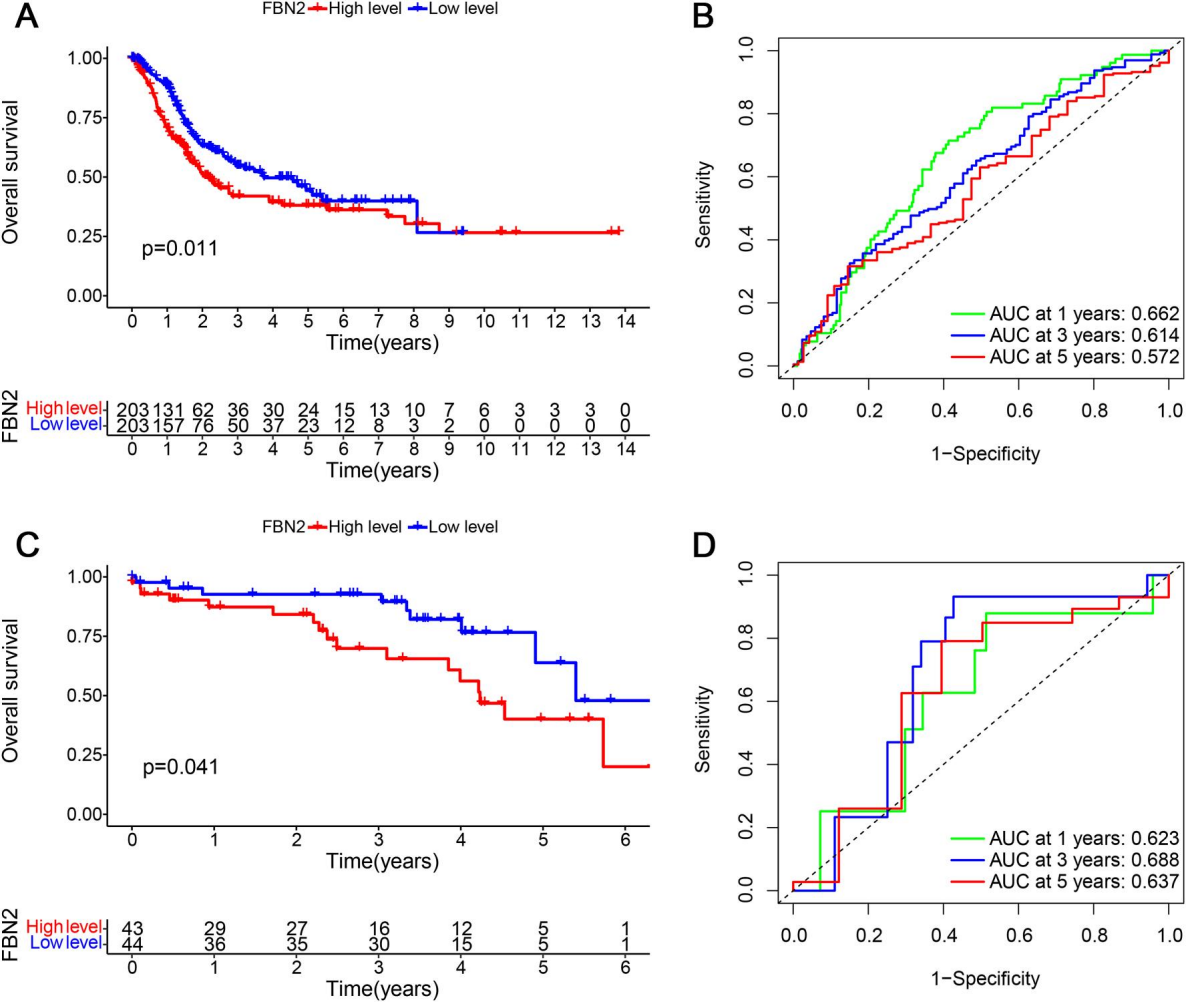
(a and c) KM curves for the high FBN2 level group and the low FBN2 level group. The table below shows the grouping situation. (b and d) ROC curves. The dotted line is the reference line (AUC = 0.5). Different colours represent the AUC calculated according to different time lengths.

The results of the KM analysis showed that the overall survival was higher in the low FBN2 expression level group than in the high FBN2 expression level group based on TCGA (*p* value = 0.011) or GSE19915 (*p* value = 0.041) (Figure 2a,c). ROC analysis was used to assess the ability to predict prognosis (Figure 2b,d). For TCGA samples, the AUCs at 1, 3, and 5 years were 0.662, 0.614, and 0.572 respectively. For GSE19915 samples, the AUCs at 1, 3, and 5 years were 0.623, 0.688, and 0.637 respectively. The results of KM analysis verified that high expression of FBN2 was associated with survival risk.

### 3.3 FBN2 is an independent prognostic factor

**FIGURE 3:**
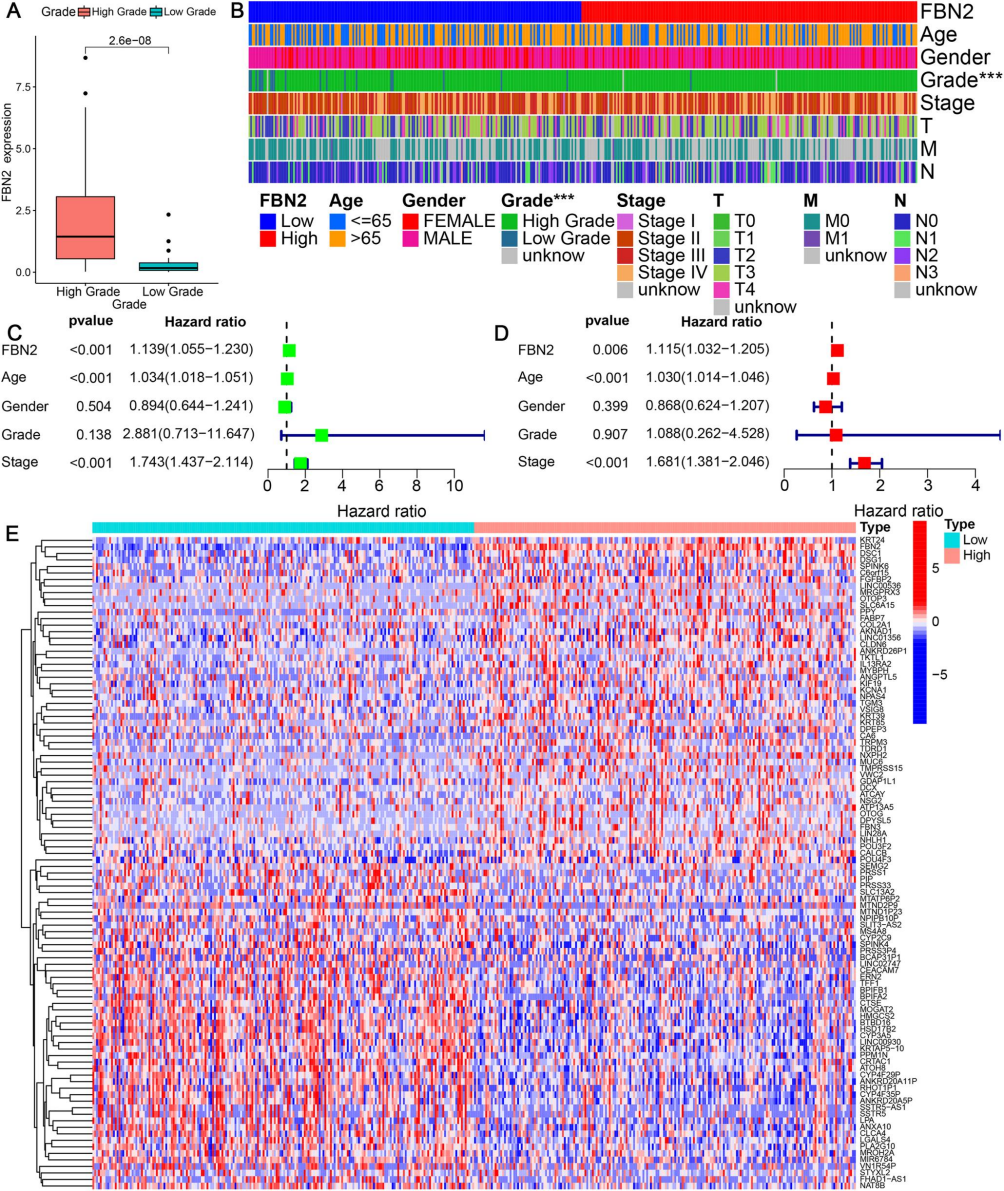
(a) Box plot for the relationship between clinical grade and FBN2 expression. The horizontal axis represents the clinical grade, and the vertical axis represents FBN2 expression. (b) Heatmap for the relationship between FBN2 and clinical features. The columns represent samples. (c) (d) Forest plots based on univariate and multivariate Cox regression. The 95% confidence intervals are shown in parentheses in the hazard ratio. (e) Heatmap for the DEGs between low and high FBN2 expression group. The columns represent samples, and the rows represent genes. The colour of each square represents the gene expression of a sample.

The results of the relationship between FBN2 expression levels and clinical features are shown in the heatmap (Figure 3b). Except for grade, FBN2 expression was not significantly associated with clinical features. Therefore, the analysis of the relationship between FBN2 expression and the clinical grade was independently performed, and the *p* value was calculated to be less than 0.001, which may suggest that FBN2 was related to grade (Figure 3a). The results of the univariate and multivariate Cox regression are shown in forest plots (Figure 3c,d). The results suggested that FBN2, age and stage could be considered independent risk factors.

### 3.4 Functions enrichment of DEGs

**FIGURE 4:**
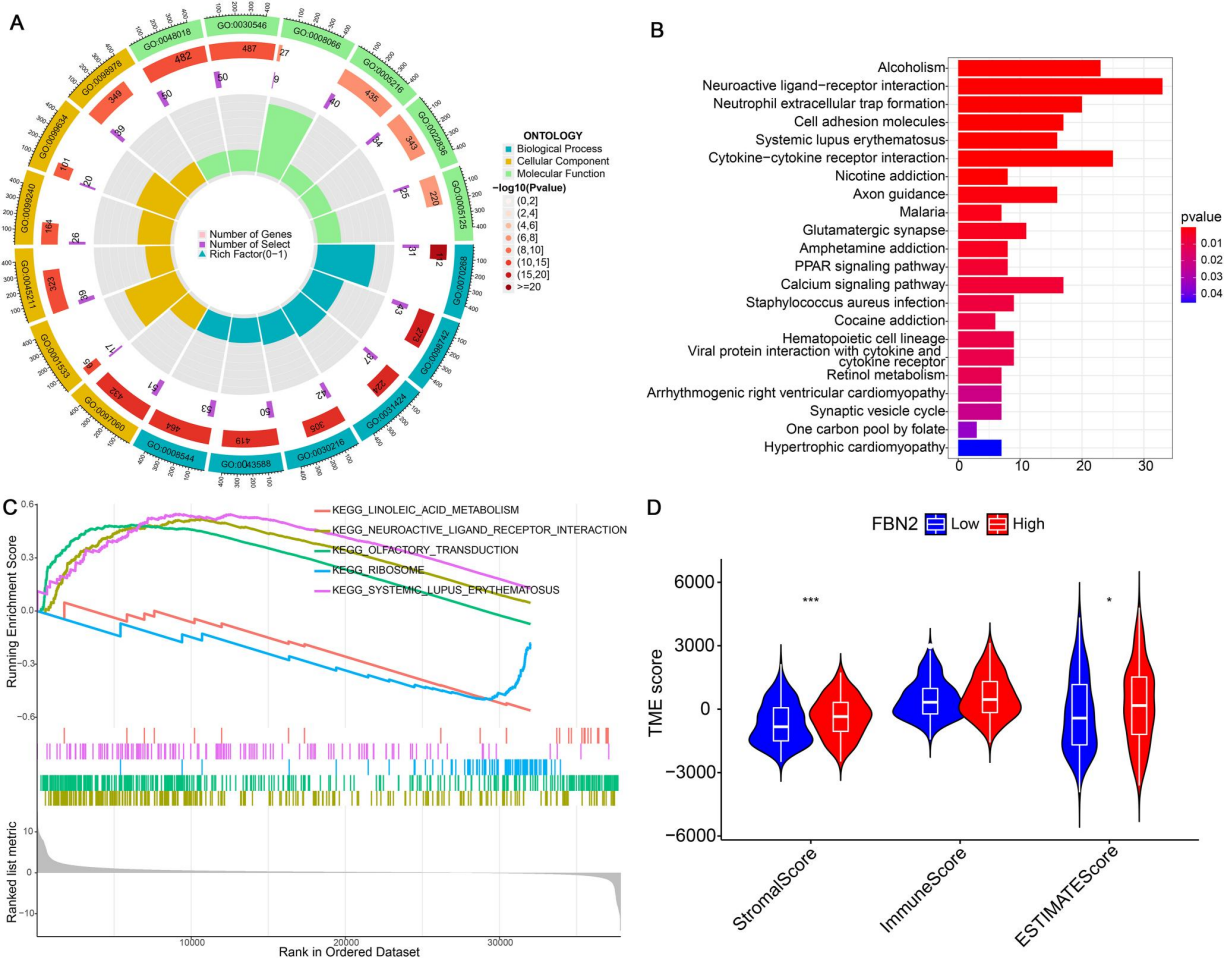
(a) Annular figure of GO analysis. The first ring shows the function IDs. The second ring shows the number of genes that were enriched in the corresponding ID, and different colours represent different *p* values. The third ring shows the number of DEGs that were enriched in the corresponding ID. (b) The results of KEGG enrichment. (c) the top three curves represent the pathways enriched based on the FBN2 high expression level group. The two curves in the bottom represent the pathways enriched based on the FBN2 low expression level group. (d) The violin plot for TME scoring analysis.

A total of 1039 DEGs were finally filtered between BLCA patients with high and low FBN2 expression, and the results are shown in a heatmap (Figure 3e). GO enrichment analysis was performed, and the first six functions in each ontology with the lowest *p* value were selected to be shown in the diagram (Figure 4a). The mapping between the GO IDs and their corresponding functions is recorded in supplementary material: table 1 (Table S1). For biological process (BP) molecular function (MF) and cellular component (CC), the DEGs were mainly associated with cornification, receptor ligand activity and synaptic membrane respectively.

KEGG enrichment analysis was also performed, and the results are shown (Figure 4b). The result of the GSEA based on KEGG enrichment suggested that the functions of DEGs in high FBN2 expression were enriched in systemic lupus erythematosus, neuroactive ligand receptor interaction, and olfactory transduction. While the functions of DEGs in low FBN2 expression were enriched in the linoleic acid metabolism and ribosome(Figure 4c).

### 3.5 Analysis of the correlation between FBN2 and the TME

Based on ESTIMATE, the stromal score, immune score, and ESTIMATE score (the sum of the stromal score and immune score) for the low FBN2 expression group and the high FBN2 expression group were calculated and compared. The high FBN2 expression level group had significantly higher stromal and ESTIMATE scores than the low expression level group (Figure 4d). The active stroma is considered immunosuppressive, and the possible reason is that the active stroma may prevent immune cells from penetrating tumour tissue [41, 42]. This may suggest that high FBN2 expression was associated with immunosuppression.

**FIGURE 5:**
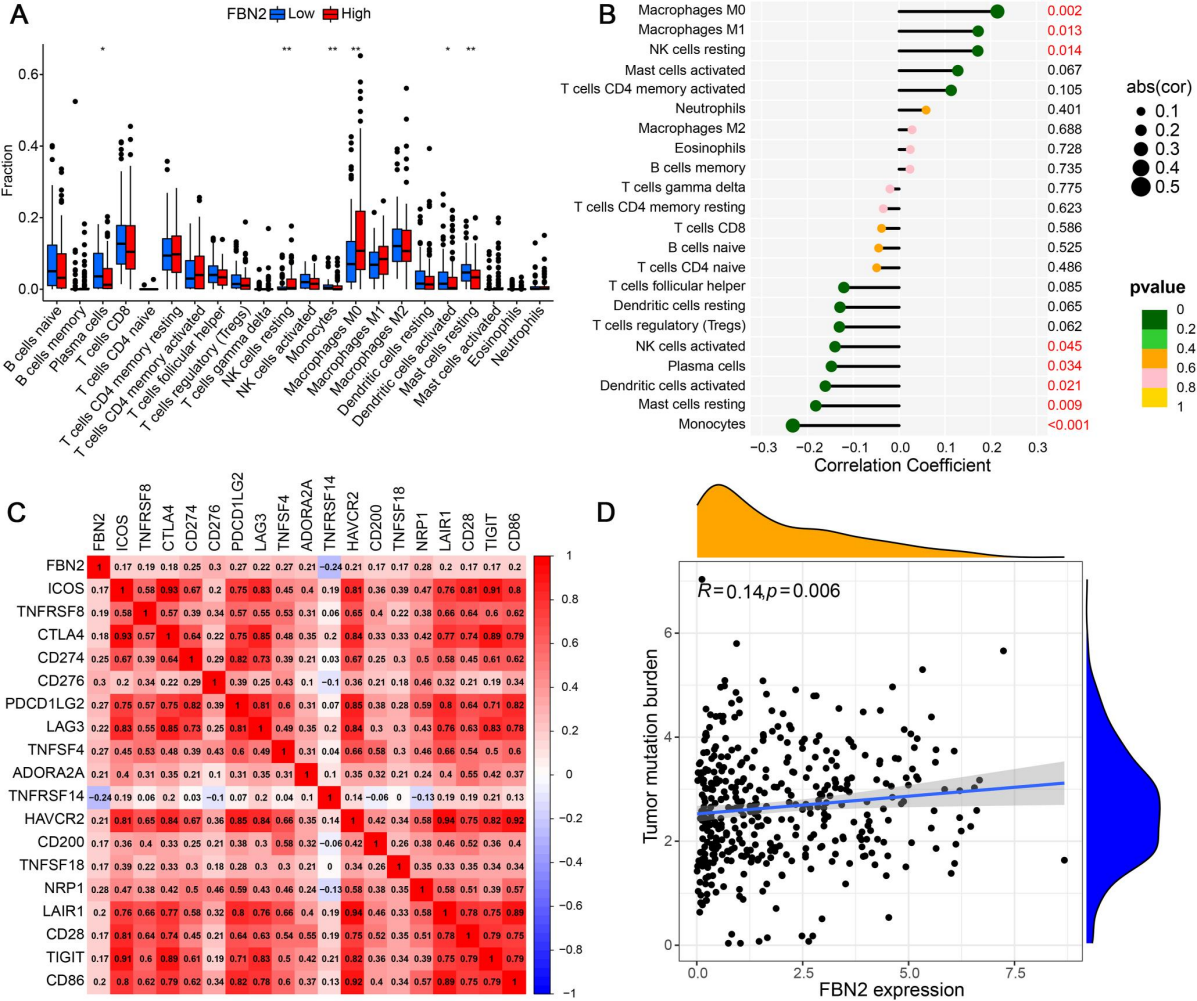
(a) Box plot for the analysis of immune cell infiltration. (b) The diagram shows the correlation between FBN2 expression and immune cells. The cells with a *p* value < 0.05 are marked in red. (c) Heatmap of the relationship between FBN2 and immune checkpoints genes. Each number and colour in the heatmap represents the correlation coefficient between the factors on the horizontal and vertical axes. (d) FBN2 expression on the horizontal axis and TMB on the vertical axis.

The difference in immune cell infiltration was explored between the high and low FBN2 expression groups (Figure 5a). Among the results that were statistical significance, we found that plasma cells, monocytes, dendritic cells activated, and mast cells resting were higher in the FBN2 low expression group, while NK cells resting and macrophages M0 were higher in the FBN2 high expression group. The correlation between immune cells and FBN2 expression was explored (Figure 5b). The results of correlation analysis with *p*‐value <0.05 were macrophages M0, M1, NK cells resting which were positively correlated with FBN2 expression, while NK cells activated, plasma cells, dendritic cells activated, mast cells resting, and monocytes which were negatively correlated with FBN2 expression. Besides, the correlation between FBN2 expression and the immune checkpoint genes was performed and the results with *p*‐value <0.001 were shown in the heatmap (Figure 5c). Except for TNFRSF14, high FBN2 expression was associated with upregulation of immune checkpoints. For the TMB, FBN2 expression was positively but weakly correlated with TMB based on Spearman statistical method with *R* = 0.14 and *P* = 0.006 (Figure 5d).

### 3.6 Susceptibility analysis for chemotherapy drugs commonly used for BLCA

**FIGURE 6:**
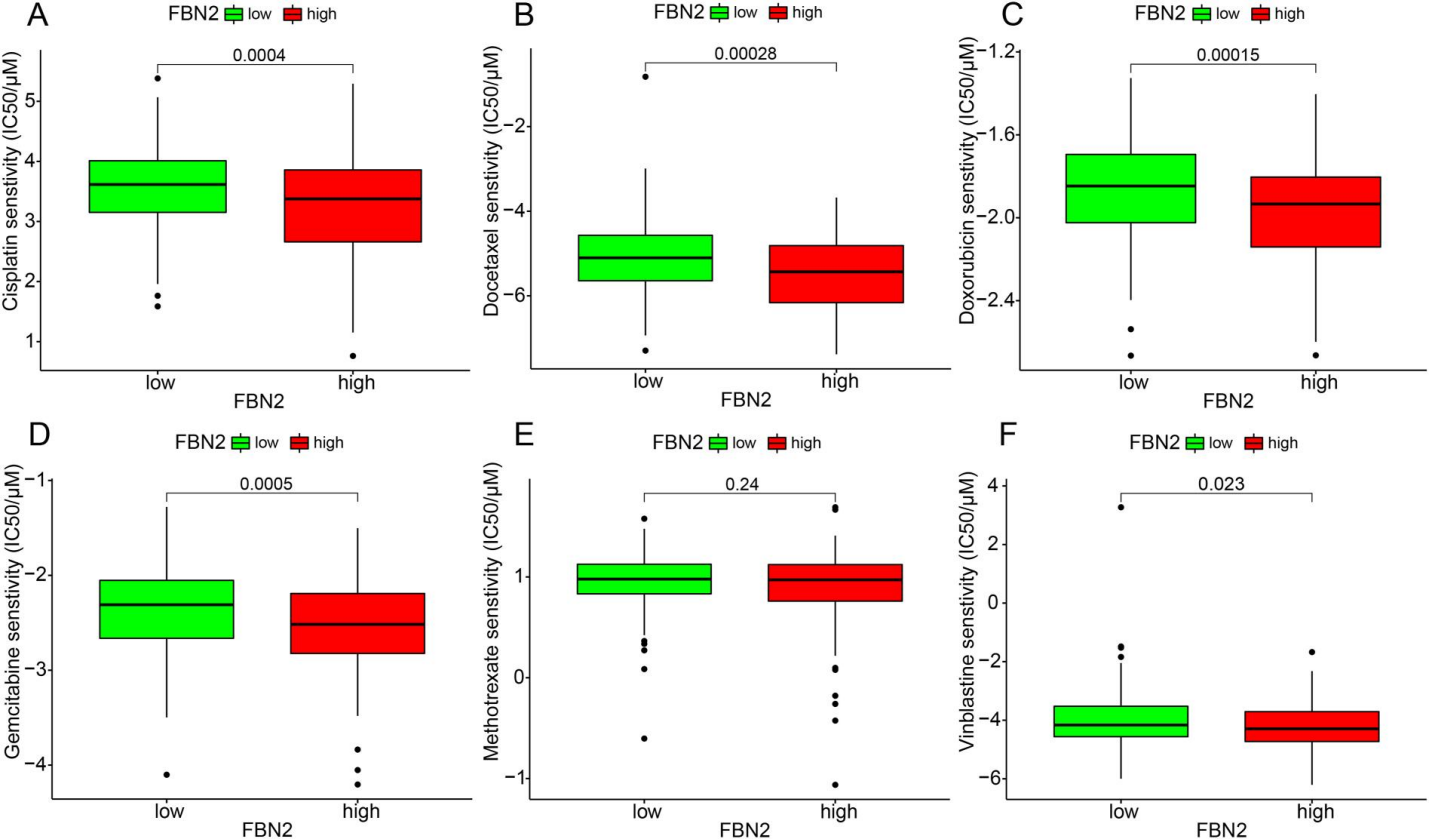
Box plots of the relationship between FBN2 and drug susceptibility of Cisplatin(A, *p* = 0.0004), Docetaxel(B, *p* = 0.00028), Doxorubicin(C, *p* = 0.00015), Gemcitabine(D, *p* = 0.0005), Methotrexate(E, *p* = 0.24), and Vinblastine(F, *p* = 0.023). The lower the IC50 is, the higher the drug sensitivity. The number between the two boxes is the *p* value.

Except for methotrexate, the chemotherapy drugs commonly used for BLCA, including cisplatin, docetaxel, doxorubicin, gemcitabine, and vinblastine, were all found to have statistically significant differences in drug susceptibility between the high and low FBN2 expression groups(Figure 6 a–f). The IC50 values of the 5 chemotherapy drugs were all lower in the high FBN2 expression group than in the low FBN2 expression group. Commonly, a lower IC50 indicates higher drug sensitivity. Therefore, the result may suggest that high FBN2 expression group was more sensitive to these chemotherapy drugs.

## 4 DISCUSSION

In our study, the Pan‐cancer analysis for FBN2 was first performed. We found that there were significant differences between some types of tumours and corresponding normal tissues, including BLCA. The data of BLCA patients and normal tissues were downloaded from TCGA and GSE19915 for follow‐up analysis. KM analysis for high and low FBN2 expression levels was performed, and the results showed that high FBN2 expression was associated with low overall survival. Moreover, we explored the correlation between FBN2 and clinical features, and the results proved that FBN2 can be a risk factor for BLCA. Thus, we demonstrate that over‐expression of FBN2 may be a risk factor for survival in patients with BLCA. The low survival rate in patients with high FBN2 expression may be related to immune escape caused by activation of the immune stroma according to the results of TME analysis. In addition, the results of our susceptibility analysis showed that high FBN2 expression was associated with the better sensitivity of five chemotherapeutic agents in the treatment of BLCA, which may suggest that these chemotherapeutic agents were more suitable for BLCA patients with high FBN2 expression.

Currently, more in‐depth research has been done on the involvement of FBN2 primarily in colon cancer. According to clinical studies, 63% of patients with primary colorectal cancer had the FBN2 gene methylated in their tumour samples; this suggests that FBN2 may be a novel diagnostic for the hepatic metastases of colorectal cancer [43]. The FBN2 has a significant potential to detect BLCA, despite the fact that little is known about its biological role in relation to epigenetic alterations in human malignancies. In our study, we discovered that FBN2 and increased FBN2 were consistently associated with shorter BLCA patient survival. This finding stimulates the notion to further explore the function of FBN2 in other domains. Similar to this, elevated FBN2 expression was discovered to be a risk factor for stomach and lung cancer [44, 45]. Additionally, it also has been demonstrated that FBN2 might serve as tumour‐suppressive effects and is a characteristic basement membrane marker in several types of cancer [46, 47]. Additionally, it has been suggested that FBN2 may be a highly effective diagnostic tool for rhabdomyosarcoma and smooth muscle sarcoma [48].

Keratins are encoded by 54 genes, which are the major subgroup of intermediate filament (IF) proteins [49]. IFs are involved in the formation of cytoskeletal systems and play an important role in maintaining the stability of epithelial tissue [50]. We found that the functions of DEGs were mainly focused on cornification in GO enrichment. UC, the major type of BLCA, is a cancer of the epithelial cells destined to be closely related to keratin. Different molecular subtypes of UC commonly have different keratin levels [51], and keratin expression profiles have been recognized as diagnostic and prognostic markers for BLCA [52]. According to recent studies, Keratin 17 (K17) targets tumour suppressors and mediates signals to promote tumour growth [53]. K17 is not only a very promising therapeutic target but is also used to improve the diagnostic accuracy of UC and detect recurrence [53, 54]. Our study suggests that FBN2 may affect cornification, but the specific regulatory mechanism still needs further study.

Natural killer (NK) cells are important innate immune cells to kill cancer cells without prior sensitisation [55, 56]. The results of our Immune cell infiltration analysis suggested that high FBN2 may lead to NK cells resting and the reason may be related to immunosuppression of the TME(55). According to the study, NK cells at rest are not hazardous to tumour cells, however NK cells that have been activated by IL‐2 or IL‐15 have the ability to prevent tumour growth [57]. Mast cell (MC) has interactions with immunosuppressive cells and is associated with inflammatory [58, 59]. MC plays complex and dynamic functions in different cancers, which may promote or inhibit cancer [59]. The macrophages in TME are called the TAMs which are related to poor prognosis [60]. The high FBN2 expression group had significantly higher expression of immunosuppressive molecules (e.g. CTLA‐4) and increased levels of various immunosuppressive cells (e.g. macrophages) than the low FBN2 expression group, suggesting that the weakened immune phenotype in the high FBN2 expression group may be due to its stronger immunosuppressive environment and immune checkpoint expression than the low FBN2 expression group. At present, the understanding of the role of TAM in TME in BLCA is limited and it is still an is a research hotspot due to its important role in the progression and metastasis of BLCA [61]. This study suggests that FBN2 may regulate the immune microenvironment through the above‐mentioned immune cells, thereby affecting the progression of bladder cancer.

In our study, patients with high levels of FBN2 expression also had greater amounts of TMB, and these two factors worked together to predict how patients with BLCA would do. A potential predictive biomarker of clinical response to immune checkpoint inhibitor treatment is TMB, or the amount of somatic missense mutations per million bases (MB) in a tumour's genes [62]. Neoantigens are produced as a result of the slow accumulation of somatic mutations, which activate T cell immunogenicity and suppress tumour cells [63]. High TMB tumours produce many antigens, making them immunogenic and triggering an anticancer response [64]. Our findings demonstrated that increased TMB tended to predict poorer survival, which was consistent with the earlier investigations.

In conclusion, we analysed the relationship between FBN2 expression and BLCA and demonstrated that FBN2 can serve as an independent prognostic factor for BLCA. We also provide new insights into the relationship between the TME and FBN2 in BLCA. Therapies targeting FBN2 may help mitigate immune escape and enhance the effectiveness of immunotherapy.

## AUTHOR CONTRIBUTION

All authors have made substantial contributions to the work: Zechao Lu, Zeguang Lu, Yongchang Lai and Haobin Zhou conceived this study; Zechao Lu, Yongchang Lai and Zhibiao Li completed the data collection; Zechao Lu, Zeguang Lu, Yongchang Lai, Haobin Zhou, Hongcheng Luo, completed the data analysis; Zechao Lu, Zeguang Lu, Yushu Chen, Jianyu Li and Jishen Zhang completed the collating of the results; Zechao Lu, Zeguang Lu, Yongchang Lai, Wanyan Cai, Zeyao Xu, participated in the completion of the manuscript; Zhaohui He and Fucai Tang supervised the study.

## CONFLICT OF INTEREST STATEMENT

All authors declare that they have no conflicts of interest with the state.

## Supporting information

Supporting Information S1

## Data Availability

The gene expression and follow‐up clinical data of BLCA patient samples were downloaded from the TCGA (https://cancergenome.nih.gov/) and GSE19915 on GEO (https://www.ncbi.nlm.nih.gov/geo).

## References

[1] Sung, H. , et al.: Global cancer statistics 2020: GLOBOCAN estimates of incidence and mortality worldwide for 36 cancers in 185 countries. CA Cancer J Clin 71(3), 209–49 (2021). 10.3322/caac.21660 33538338

[2] Kamat, A.M. , et al.: Bladder cancer. Lancet 388(10061), 2796–810 (2016). 10.1016/s0140-6736(16)30512-8 27345655

[3] Nadal, R. , Bellmunt, J. : Management of metastatic bladder cancer. Cancer Treat Rev. 76, 10–21 (2019). 10.1016/j.ctrv.2019.04.002 31030123

[4] Hatogai, K. , Sweis, R.F. : The tumor microenvironment of bladder cancer. Adv. Exp. Med. Biol. 1296, 275–90 (2020) 34185299 10.1007/978-3-030-59038-3_17 PMC8345230

[5] Kamat, A.M. , et al.: Expert consensus document: consensus statement on best practice management regarding the use of intravesical immunotherapy with BCG for bladder cancer. Nat. Rev. Urol. 12(4), 225–35 (2015). 10.1038/nrurol.2015.58 25800393

[6] Chehroudi, A.C. , Black, P.C. : Emerging intravesical therapies for the management of bacillus Calmette‐Guerin (BCG)‐unresponsive non‐muscle‐invasive bladder cancer: charting a path forward. Can Urol Assoc J 14(6), 204–13 (2020). 10.5489/cuaj.6101 31977307 PMC7654668

[7] Han, J. , et al.: Mechanisms of BCG in the treatment of bladder cancer‐current understanding and the prospect. Biomed. Pharmacother. 129, 110393 (2020). 10.1016/j.biopha.2020.110393 32559616

[8] Lopez‐Beltran, A. , et al.: Immune checkpoint inhibitors for the treatment of bladder cancer. Cancers 13(1), 131 (2021). 10.3390/cancers13010131 33401585 PMC7795541

[9] Funt, S.A. , Rosenberg, J.E. : Systemic, perioperative management of muscle‐invasive bladder cancer and future horizons. Nat. Rev. Clin. Oncol. 14(4), 221–34 (2017). 10.1038/nrclinonc.2016.188 27874062 PMC6054138

[10] Gakis, G. , et al.: Systematic review on the fate of the remnant urothelium after radical cystectomy. Eur. Urol. 71(4), 545–57 (2017). 10.1016/j.eururo.2016.09.035 27720534 PMC5533286

[11] Patnaik, A. , et al.: Cabozantinib eradicates advanced murine prostate cancer by activating antitumor innate immunity. Cancer Discov. 7(7), 750–65 (2017). 10.1158/2159-8290.cd-16-0778 28274958 PMC5501767

[12] Yang, Y. , et al.: PTEN loss promotes intratumoral androgen synthesis and tumor microenvironment remodeling via aberrant activation of RUNX2 in castration‐resistant prostate cancer. Clin. Cancer Res. 24(4), 834–46 (2018). 10.1158/1078-0432.ccr-17-2006 29167276 PMC5816982

[13] Frederic, M.Y. , et al.: The FBN2 gene: new mutations, locus‐specific database (Universal Mutation Database FBN2), and genotype‐phenotype correlations. Hum. Mutat. 30(2), 181–90 (2009). 10.1002/humu.20794 18767143

[14] Kloth, K. , et al.: Severe congenital contractural arachnodactyly caused by biallelic pathogenic variants in FBN2. Eur. J. Med. Genet. 64(3), 104161 (2021). 10.1016/j.ejmg.2021.104161 33571691

[15] Kamal, Y. , et al.: Transcriptomic differences between primary colorectal adenocarcinomas and distant metastases reveal metastatic colorectal cancer subtypes. Cancer Res. 79(16), 4227–41 (2019). 10.1158/0008-5472.can-18-3945 31239274 PMC6697603

[16] Chen, H. , et al.: Aberrant methylation of FBN2 in human non‐small cell lung cancer. Lung Cancer 50(1), 43–9 (2005). 10.1016/j.lungcan.2005.04.013 15951052

[17] van Loon, K. , et al.: Role of fibrillin‐2 in the control of TGF‐beta activation in tumor angiogenesis and connective tissue disorders. Biochim. Biophys. Acta Rev. Canc 1873(2), 188354 (2020). 10.1016/j.bbcan.2020.188354 32119940

[18] Lind, H. , et al.: Dual targeting of TGF‐beta and PD‐L1 via a bifunctional anti‐PD‐L1/TGF‐betaRII agent: status of preclinical and clinical advances. J. Immunother. Cancer 8(1), e000433 (2020). 10.1136/jitc-2019-000433 32079617 PMC7057416

[19] Caja, L. , et al.: TGF‐Beta and the tissue microenvironment: relevance in fibrosis and cancer. Int. J. Mol. Sci. 19(5), 1294 (2018). 10.3390/ijms19051294 29701666 PMC5983604

[20] Xu, C. , et al.: Identification of a novel tumor microenvironment prognostic signature for bladder urothelial carcinoma. Front. Oncol. 12, 818860 (2022). 10.3389/fonc.2022.818860 35299749 PMC8921452

[21] Cao, R. , et al.: An EMT‐related gene signature for the prognosis of human bladder cancer. J. Cell Mol. Med. 24(1), 605–17 (2020). 10.1111/jcmm.14767 31657881 PMC6933372

[22] Li, T. , et al.: TIMER: a web server for comprehensive analysis of tumor‐infiltrating immune cells. Cancer Res. 77(21), e108–e10 (2017). 10.1158/0008-5472.can-17-0307 29092952 PMC6042652

[23] Ritchie, M.E. , et al.: Limma powers differential expression analyses for RNA‐sequencing and microarray studies. Nucleic Acids Res. 43(7), e47 (2015). 10.1093/nar/gkv007 25605792 PMC4402510

[24] Gu, Z. , et al.: Circlize Implements and enhances circular visualization in R. Bioinformatics 30(19), 2811–2 (2014). 10.1093/bioinformatics/btu393 24930139

[25] Blanche, P. , Dartigues, J.F. , Jacqmin‐Gadda, H. : Estimating and comparing time‐dependent areas under receiver operating characteristic curves for censored event times with competing risks. Stat. Med. 32(30), 5381–97 (2013). 10.1002/sim.5958 24027076

[26] Gu, Z. , Eils, R. , Schlesner, M. : Complex heatmaps reveal patterns and correlations in multidimensional genomic data. Bioinformatics 32(18), 2847–9 (2016). 10.1093/bioinformatics/btw313 27207943

[27] The Gene Ontology, C. : The gene ontology resource: 20 years and still GOing strong. Nucleic Acids Res. 47(D1), D330–D8 (2019). 10.1093/nar/gky1055 30395331 PMC6323945

[28] Kanehisa, M. , et al.: KEGG: integrating viruses and cellular organisms. Nucleic Acids Res. 49(D1), D545–D51 (2021). 10.1093/nar/gkaa970 33125081 PMC7779016

[29] Yu, G. , et al.: clusterProfiler: an R package for comparing biological themes among gene clusters. OMICS 16(5), 284–7 (2012). 10.1089/omi.2011.0118 22455463 PMC3339379

[30] Valero‐Mora, P.M. : ggplot2: elegant graphics for data analysis. J. Stat. Software 35(Book Review 1), 1–3 (2010). 10.18637/jss.v035.b01

[31] Yoshihara, K. , et al.: Inferring tumour purity and stromal and immune cell admixture from expression data. Nat. Commun. 4(1), 2612 (2013). 10.1038/ncomms3612 24113773 PMC3826632

[32] Zhang, Z. : Reshaping and aggregating data: an introduction to reshape package. Ann. Transl. Med. 4(4), 78 (2016) 27004225 10.3978/j.issn.2305-5839.2016.01.33 PMC4779770

[33] Newman, A.M. , et al.: Robust enumeration of cell subsets from tissue expression profiles. Nat. Methods 12(5), 453–7 (2015). 10.1038/nmeth.3337 25822800 PMC4739640

[34] Chan, T.A. , et al.: Development of tumor mutation burden as an immunotherapy biomarker: utility for the oncology clinic. Ann. Oncol. 30(1), 44–56 (2019). 10.1093/annonc/mdy495 30395155 PMC6336005

[35] Geeleher, P. , Cox, N. , Huang, R.S. : pRRophetic: an R package for prediction of clinical chemotherapeutic response from tumor gene expression levels. PLoS One 9(9), e107468 (2014). 10.1371/journal.pone.0107468 25229481 PMC4167990

[36] Ma, X. , et al.: Development and validation of a novel ferroptosis‐related LncRNA signature for predicting prognosis and the immune landscape features in uveal melanoma. Front. Immunol. 13, 922315 (2022). 10.3389/fimmu.2022.922315 35774794 PMC9238413

[37] Sun, J.X. , et al.: A four‐cell‐senescence‐regulator‐gene prognostic index verified by genome‐wide CRISPR can depict the tumor microenvironment and guide clinical treatment of bladder cancer. Front. Immunol. 13, 908068 (2022). 10.3389/fimmu.2022.908068 35898492 PMC9312376

[38] Yue, Z. , Sun, J. , Shi, L. : Construction and validation of a 6‐ferroptosis related gene signature for prognosis and immune landscape prediction in melanoma. Front. Genet. 13, 887542 (2022). 10.3389/fgene.2022.887542 35692844 PMC9174666

[39] Zhu, K. , et al.: Identification of a novel PPAR signature for predicting prognosis, immune microenvironment, and chemotherapy response in bladder cancer. PPAR Res. 2021, 7056506–17 (2021). 10.1155/2021/7056506 35027921 PMC8749226

[40] Garnett, M.J. , et al.: Systematic identification of genomic markers of drug sensitivity in cancer cells. Nature 483(7391), 570–5 (2012). 10.1038/nature11005 22460902 PMC3349233

[41] Chen, D.S. , Mellman, I. : Elements of cancer immunity and the cancer‐immune set point. Nature 541(7637), 321–30 (2017). 10.1038/nature21349 28102259

[42] Shao, W. , et al.: The pyroptosis‐related signature predicts prognosis and indicates immune microenvironment infiltration in gastric cancer. Front. Cell Dev. Biol. 9, 676485 (2021). 10.3389/fcell.2021.676485 34179006 PMC8226259

[43] Hibi, K. , et al.: FBN2 methylation is detected in the serum of colorectal cancer patients with hepatic metastasis. Anticancer Res. 32(10), 4371–4 (2012) 23060561

[44] Song, W. , et al.: A novel prognostic model based on epithelial‐mesenchymal transition‐related genes predicts patient survival in gastric cancer. World J. Surg. Oncol. 19(1), 216 (2021). 10.1186/s12957-021-02329-9 34281542 PMC8290588

[45] Zhang, S. , et al.: Identification of seven‐gene marker to predict the survival of patients with lung adenocarcinoma using integrated multi‐omics data analysis. J. Clin. Lab. Anal. 36(2), e24190 (2022). 10.1002/jcla.24190 34951053 PMC8841135

[46] Wang, J. , et al.: Cancer‐associated stromal fibroblast‐derived transcriptomes predict poor clinical outcomes and immunosuppression in colon cancer. Pathol. Oncol. Res. 28, 1610350 (2022) 35991839 10.3389/pore.2022.1610350 PMC9385976

[47] Chu, L. , et al.: Induction of senescence‐associated secretory phenotype underlies the therapeutic efficacy of PRC2 inhibition in cancer. Cell Death Dis. 13(2), 155 (2022). 10.1038/s41419-022-04601-6 35169119 PMC8847585

[48] Gu, H.Y. , et al.: Risk score based on expression of five novel genes predicts survival in soft tissue sarcoma. Aging (Albany NY) 12(4), 3807–27 (2020). 10.18632/aging.102847 32084007 PMC7066896

[49] Herrmann, H. , et al.: Intermediate filaments: primary determinants of cell architecture and plasticity. J. Clin. Invest. 119(7), 1772–83 (2009). 10.1172/jci38214 19587452 PMC2701873

[50] Moll, R. , Divo, M. , Langbein, L. : The human keratins: biology and pathology. Histochem. Cell Biol. 129(6), 705–33 (2008). 10.1007/s00418-008-0435-6 18461349 PMC2386534

[51] Sjodahl, G. , et al.: A molecular taxonomy for urothelial carcinoma. Clin. Cancer Res. 18(12), 3377–86 (2012). 10.1158/1078-0432.ccr-12-0077-t 22553347

[52] Zupančič, D. , Romih, R. : Immunohistochemistry as a paramount tool in research of normal urothelium, bladder cancer and bladder pain syndrome. Eur. J. Histochem. : EJH. 65(2) (2021). 10.4081/ejh.2021.3242 PMC8033529 33764020

[53] Baraks, G. , et al.: Dissecting the oncogenic roles of keratin 17 in the hallmarks of cancer. Cancer Res. 82(7), 1159–66 (2022). 10.1158/0008-5472.can-21-2522 34921015 PMC9016724

[54] Babu, S. , et al.: Keratin 17 is a sensitive and specific biomarker of urothelial neoplasia. Mod. Pathol. 32(5), 717–24 (2019). 10.1038/s41379-018-0177-5 30443013

[55] Terren, I. , et al.: NK cell metabolism and tumor microenvironment. Front. Immunol. 10, 2278 (2019). 10.3389/fimmu.2019.02278 31616440 PMC6769035

[56] Myers, J.A. , Miller, J.S. : Exploring the NK cell platform for cancer immunotherapy. Nat. Rev. Clin. Oncol. 18(2), 85–100 (2021). 10.1038/s41571-020-0426-7 32934330 PMC8316981

[57] Ma, Y. , et al.: Exploring the pathological mechanism of bladder cancer based on tumor mutational burden analysis. BioMed Res. Int. 2019, 1093815–9 (2019). 10.1155/2019/1093815 31534952 PMC6732589

[58] Rigoni, A. , Colombo, M.P. , Pucillo, C. : Mast cells, basophils and eosinophils: from allergy to cancer. Semin. Immunol. 35, 29–34 (2018). 10.1016/j.smim.2018.02.001 29428698

[59] Frossi, B. , et al.: Exploring a regulatory role for mast cells: 'MCregs. Trends Immunol. 31(3), 97–102 (2010). 10.1016/j.it.2009.12.007 20149743

[60] Singh, S. , et al.: Initiative action of tumor‐associated macrophage during tumor metastasis. Biochim Open 4, 8–18 (2017). 10.1016/j.biopen.2016.11.002 29450136 PMC5801826

[61] Rubio, C. , et al.: Toward tumor fight and tumor microenvironment remodeling: PBA induces cell cycle arrest and reduces tumor hybrid cells' pluripotency in bladder cancer. Cancers 14(2), 287 (2022). 10.3390/cancers14020287 35053451 PMC8773853

[62] Merino, D.M. , et al.: Establishing guidelines to harmonize tumor mutational burden (TMB): in silico assessment of variation in TMB quantification across diagnostic platforms: phase I of the Friends of Cancer Research TMB Harmonization Project. J Immunother Cancer 8(1), e000147 (2020). 10.1136/jitc-2019-000147 32217756 PMC7174078

[63] Li, D. , et al.: Comprehensive analysis of cuproptosis‐related lncRNAs in the prognosis and therapy response of patients with bladder cancer. Ann. Transl. Med. 10(22), 1232 (2022). 10.21037/atm-22-5294 36544685 PMC9761144

[64] Wu, Z. , et al.: Identification of gene expression profiles and immune cell infiltration signatures between low and high tumor mutation burden groups in bladder cancer. Int. J. Med. Sci. 17(1), 89–96 (2020). 10.7150/ijms.39056 31929742 PMC6945555

